# Continuous LVAD monitoring reveals high suction rates in clinically stable outpatients

**DOI:** 10.1111/aor.13638

**Published:** 2020-03-01

**Authors:** Christoph Gross, Heinrich Schima, Thomas Schlöglhofer, Kamen Dimitrov, Martin Maw, Julia Riebandt, Dominik Wiedemann, Daniel Zimpfer, Francesco Moscato

**Affiliations:** ^1^ Center for Medical Physics and Biomedical Engineering Medical University of Vienna Vienna Austria; ^2^ Ludwig Boltzmann Institute Cardiovascular Research Vienna Austria; ^3^ Division of Cardiac Surgery Department of Surgery Medical University of Vienna Vienna Austria

**Keywords:** left ventricular assist device, mechanical circulatory support, outpatient monitoring, overpumping, suction, tachycardia

## Abstract

Suction of the left ventricle can lead to potentially life‐threatening events in left ventricular assist device (LVAD) patients. With the resolution of currently available clinical LVAD monitoring healthcare professionals are unable to evaluate patients’ suction occurrences in detail. This study investigates occurrences and durations of suction events and their associations with tachycardia in stable outpatients. Continuous high‐resolution LVAD data from HVAD patients were analyzed in the early outpatient period for 15 days. A validated suction detection from LVAD signals was used. Suction events were evaluated as suction rates, bursts of consecutive suction beats, and clusters of suction beats. The occurrence of tachycardia was analyzed before, during, and after suction clusters. Furthermore, blood work, implant strategy, LVAD speed setting, inflow cannula position, left ventricular diameters, and adverse events were evaluated in these patients. LVAD data of 10 patients was analyzed starting at 78 ± 22 postoperative days. Individuals’ highest suction rates per hour resulted in a median of 11% (range 3%‐61%). Bursts categorized as consecutive suction beats with n = 2, n = 3‐5, n = 6‐15, and n > 15 beats were homogenously distributed with 10.3 ± 0.8% among all suction beats. Larger suction bursts were followed by shorter suction‐free periods. Tachycardia during suction occurred in 12% of all suction clusters. Significant differences in clinical parameters between individuals with high and low suction rates were only observed in left ventricular end‐diastolic and end‐systolic diameters (*P* < .02). Continuous high‐resolution LVAD monitoring sheds light on outpatient suction occurrences. Interindividual and intraindividual characteristics of longitudinal suction rates were observed. Longer suction clusters have higher probabilities of tachycardia within the cluster and more severe types of suction waveforms. This work shows the necessity of improved LVAD monitoring and the implementation of an LVAD speed control to reduce suction rates and their concomitant burden on the cardiovascular system.

## 1 INTRODUCTION

For patients with terminal heart failure, left ventricular assist devices (LVAD) are increasingly used as a bridge to cardiac transplantation or even as a destination therapy, if no donor organ is available or a transplant is contraindicated.^1^ Currently, implanted LVADs operate at constant speeds; some devices perform a predefined temporary speed modulation periodically.^2^, ^3^ Before hospital discharge, pump speed is individually adjusted to provide sufficient unloading of the left ventricle while restoring adequate systemic blood flow simultaneously.^4^ During outpatient care visits, LVAD speed might be re‐adjusted by healthcare professionals according to the patient's condition. LVADs, operating at fixed speeds, offer limited adaption to generate pump flow according to the changing physiological demands, rather delegating this control task to the sick heart and its remaining native responsive mechanisms.^5^ During particular hemodynamic conditions, the LVAD pumps more blood than delivered from the right ventricle, which results in continuously decreasing left ventricular dimensions until the LVAD’s inflow cannula is occluded by suction of the septum or the lateral wall. This occlusion temporarily reduces pump flow, creates a jetstream with high shear stress, damages blood components, and may create hematoma or even necrosis and erosion in the mechanically stressed endocardial layers.^6^, ^7^ Suction activates platelets and increases hemolysis as well as the risk of subsequent thrombogenicity.^8^, ^9^, ^10^ The mechanical irritation of the endocardium can also lead to rhythm disorders.^11^, ^12^, ^13^ Therefore, to provide adequate LVAD support, suction events should be avoided.

Currently available LVAD systems are equipped with alarm algorithms to recognize the occurrence of severe suction events. Because of poor resolution, the pumps’ log data stored in the LVAD controllers provide insufficient information for healthcare professionals for the retrospective evaluation of events with short durations. Examples for HVAD log data snapshots are provided in references.^14^, ^15^ Additionally, the HVAD system (Medtronic Inc, Minneapolis, MN, USA) has provided visualization of the instantaneous estimated pump flow on an external cable connected monitor, which allows visual inspection of the flow waveforms during hospital visits or outpatient follow‐ups. This real‐time monitoring of the estimated LVAD flow waveforms allows experienced clinicians to diagnose suction by recognition of the distinct waveforms.^16^ This ability to detect suction waveforms visually was further improved by a recent controller update that introduced a flow estimator with increased frequency bandwidth.^17^

To improve the currently available LVAD logs, a data recorder was developed in our center, which allows uninterrupted 24/7 recording for several weeks and analysis of flow waveforms. Based on this continuous data recording, indices characterizing the assisted cardiac circulation can be calculated such as average, diastolic and systolic LVAD flow rates,^17^ heartrate,^18^ incidence of aortic valve opening,^19^, ^20^ and parameters for contractility^21^ and relaxation.^22^

The LVAD data recorder used in this study allowed obtaining the high‐resolution insight into pump performance of outpatients for the first time. These recordings provide necessary information to detect abnormal behavior of the patients’ hemodynamic condition remotely. The aim of this study is to evaluate suction occurrences and consequences for heart rhythm disorders of VAD outpatients judged as clinically stable.

## 2 PATIENTS AND METHODS

Data from LVAD patients enrolled in a clinical study for continuous LVAD monitoring were analyzed retrospectively for a defined early outpatient period of 15 days in regards to suction events and tachycardia.

### 2.1 Clinical study

In an observational study, a proprietary data recorder, which allows the continuous storage of beat‐to‐beat pump signals for several weeks, was applied to 38 LVAD‐patients between June 1, 2014 and March 1, 2016. These selected patients were compliant to manage their LVAD peripherals, able to follow their therapeutic protocol, with age >18 years and gave written consent to participate in the study approved by the Institutional Review Board of the Medical University Vienna (EK‐243/2011, ClinicalTrials.gov identifier: NCT01981642).

**Figure 1:**
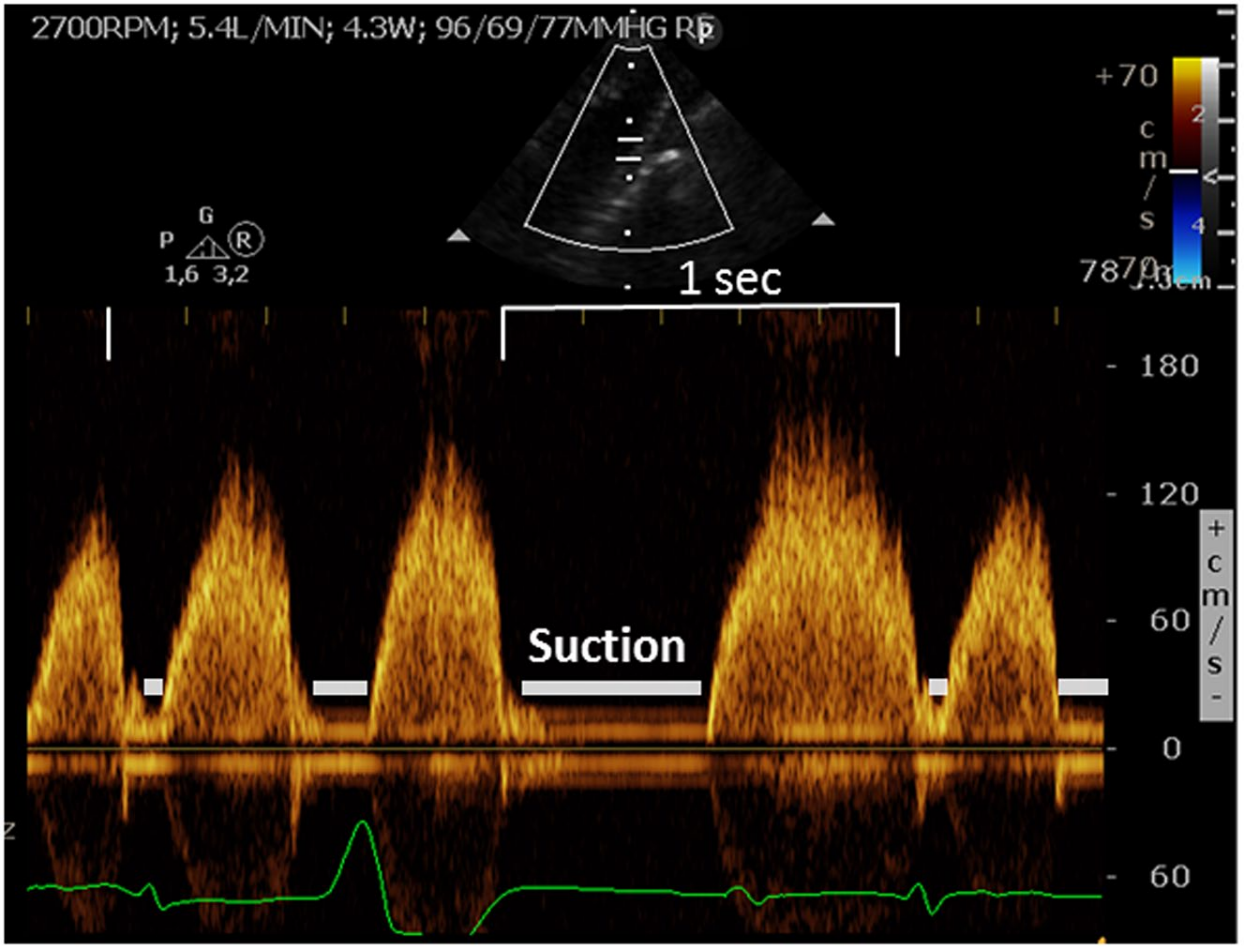
Pulsed‐wave Doppler interrogation of the outflow graft by transthoracic echocardiography in LVAD patient. The spectral Doppler waveform shows abrupt intermittent interruptions of the Doppler signal (marked with white horizontal bars) due to intermittent suction. During these suction events (depicted by the white bars) abnormally low diastolic velocities were measured for the continuous flow LVAD with a speed of 2700 rpm [Color figure can be viewed at wileyonlinelibrary.com]

To investigate incidence of suction in clinically stable outpatients, a subset of patients was analyzed which received the recorder immediately after discharge from Phase‐II (postoperative and inpatient) cardiac rehabilitation. Rehabilitation included appropriate pump adjustment, optimization of pharmacologic therapy, fluid balance, training to restore patients' independence during daily life, and to achieve confidence in handling of LVAD peripherals within a period of 4 weeks.^23^ All patients with at least 15 days of continuous LVAD data recorded from the first hospital follow‐up immediately after cardiac rehabilitation were included. Patients with only sparse amount of LVAD data available during the observation period were excluded from analysis. Therefore, the selected group of patients was considered to have been optimally adjusted, trained, motivated to regain independence in daily living, and without any adverse events during the observation period. At the beginning of the observation period, blood markers such as LDH (lactate dehydrogenase) and proBNP were assessed during the outpatient follow‐up. The transthoracic echocardiography recording closest to the observation period was analyzed from each patient. The position of the inflow cannula within the left ventricle was qualitatively classified based on the echocardiographer's subjective opinion as either optimal (central position within the left ventricle and quasi perpendicular axis to the mitral valve plane) or suboptimal (large angulation of the cannula's axis to the mitral valve plane, axis toward the ventricular wall, or a direct proximity to the endocardial surface with or without visualization of suction of endocardial surface). Suction during the echocardiographic examination was assessed by Doppler interrogation of the outflow graft and classified qualitatively by an abrupt partial or complete interruption of the spectral Doppler velocity waveform with abnormal low or even unmeasurable diastolic velocities compared to reference values provided in the articles.^24^, ^25^ An echocardiographic illustration for the spectral Doppler measurement of the outflow graft with suction is shown in Figure 1. Furthermore, patient occurrences of adverse events were analyzed from medical records.

### 2.2 LVAD monitoring

Our group developed a miniaturized data recorder, which records the continuous data stream provided by the HVAD (Medtronic Inc, Minneapolis, MN, USA) serial monitor port. With a size of only 80 × 24 × 48 mm, the battery powered LVAD data recorder fits within the patients’ carriage bag for LVAD peripherals. Monitored LVAD data (LVAD impeller speed, motor current, and power uptake) were recorded at a rate of 50 samples/sec and stored on a microSD card. Pump flow was estimated based on a high‐frequency algorithm^17^ and parameters such as mean flow, pulsatility, and heart rate^18^ were calculated using MATLAB (TheMathworks Inc, Natick, MA, USA).

### 2.3 Suction detection

A beatwise suction detection based on 16 already reported time domain features^26^, ^27^, ^28^, ^29^, ^30^, ^31^ derived from the estimated pump flow,^17^ LVAD motor current and impeller rotational speed was developed using a bagged decision tree classifier.^32^ A database of 500 LVAD waveform snapshots with 10‐second duration from all patients of the continuous LVAD monitoring study (n = 38) was established and classified beat‐by‐beat by six experts. The valid expert pooled decisions resulted in a ratio of 61/39% nonsuction/suction beats in this database. Training and validation of the classifier was performed with leave‐one‐out cross‐validation.^33^ Compared to the pooled experts' opinion the suction classifier had a sensitivity of 96.8%, specificity of 98.2% and an accuracy of 97.7%. *(It should be noted, that this accuracy was based on the snapshots with a balanced number of suction/nonsuction waveforms and its intentionally high percentage of critical examples observed in patients.)*

The beatwise suction detection was applied on the patient data obtained of the 15 days observation period. Beats during the periodic Lavare speed modification cycle were excluded. Three types of analyses were performed: 1. Suction rates, 2. Suction bursts, and 3. Clusters of suction events and correlated tachycardia.

### 2.4 Suction rates

Results were calculated as percentage of beats with detected suction compared to all heart beats in non‐overlapping time windows of either 1 hour, 1 day, or over the whole observation period. The results were investigated for each patient as longitudinal profiles and circadian patterns (averaged over 15 days). The patients' maximum suction rates per hour and number of occurrences of suction rates ≥5%/hour were analyzed using descriptive statistics and linear Pearson correlation. The suction rate is an easily calculated and interpreted metric containing no information about the distribution of the occurred suction beats within the time window.

### 2.5 Suction bursts

Suction bursts were defined as a number of consecutive beats with suction detected. Bursts were grouped into four classes, depending on the amount of subsequent suction beats (n = 2, n = 3‐5, n = 6‐15, and n ≥ 15). Associations of suction‐free periods after suction bursts according to the preceding burst size were analyzed with linear Pearson correlation. Suction bursts include temporal information within the windows due to the evaluation of sequences with only suction beats.

### 2.6 Clusters of suction events and correlated tachycardia

Suction clusters were defined as occurrences of suction events or bursts with suction‐to‐suction time intervals of less than 10 seconds. Recurrence of further suction events extended the duration of a cluster. Suction clusters were analyzed in subgroups based on durations (≤1 minute, >1 & ≤5 minute, and >5 minutes). Tachycardia was defined as 5 or more subsequent occurring cardiac cycles with a beat‐to‐beat interval <600 msec (>100 bpm) calculated from the estimated pump flow.^34^ Beat‐to‐beat intervals with flow pulsatility <0.7 L/min were excluded from analysis, as low flow pulsatilities make the detection of the heart rate unreliable. Tachycardia was evaluated for periods of <10 seconds before, within and <10 seconds after suction clusters. Suction clusters represent episodes consisting of frequent as well as infrequent suction events connected to each other. Therefore, the identified suction clusters may vary in durations and suction rates.

### 2.7 Statistical analysis

Data were processed and analyzed with MATLAB. Metric variables are reported as mean ± standard deviation (SD) for normally distributed data and as median with range for nonnormally distributed data. Normal distribution of metric variables was determined with the Shapiro‐Wilk test. The Student's *t*‐test was used to determine statistical significance for normal distributed data and the Wilcoxon signed rank test for nonparametric variables. For the cluster analysis, results (median and range) were calculated from the patients' median levels.

## 3 RESULTS

### 3.1 Patient demographics

Of the 38 patients within the observational study for continuous LVAD monitoring 10 fulfilled the requirements described above, that is, available data for at least 15 days immediately after discharge from Phase II rehabilitation and without readmission during the observation period. Demographics of these 10 patients are given in Table 1. LVAD data from the study patients were available for 97% of the observation period (3% were missing due to short interruptions, resynchronizations, or temporary disconnections of the recording device).

**Table 1:** Patient characteristics (n = 10). Interagency registry for mechanically assisted circulatory support (Intermacs), extracorporeal membrane oxygenation (ECMO), implantable cardioverter defibrillator (ICD), pacemaker (PM), cardiac resynchronization therapy (CRT)

	Mean ± SD	n
Age (years)	58 ± 9.6	
Gender		M: 8 (80%), M: 2 (20%)
Weight (kg)	81 ± 15	
Height (cm)	178 ± 8	
BMI (kg/m^2^)	25 ± 3.3	
Etiology of cardiomyopathy		Dilated: 5 (50%)
		Ischemic: 5 (50%)
INTERMACS level		1:2 (20%)
		2:3 (30%)
		3:3 (30%)
		4:2 (20%)
Intraoperative bypass support		CPB: 5 (50%)
		ECMO: 1 (10%)
		Off‐pump: 4 (40%)
Operation technique		Sternotomy: 3 (30%)
		Hemi‐sternotomy & thoracotomy: 1 (10%)
		Bilateral thoracotomy: 6 (60%)
Outflow graft location		Subclavian artery: 4 (40%)
		Aorta ascendens: 6 (60%)
Pacemaker/Defibrillator		ICD: 3 (30%)
		ICD + PM: 2 (20%)
		ICD + CRT: 1 (10%)
Initial length of hospital stay (days)	38 ± 9.8	
Begin of the observation period (postoperative days)	78 ± 22	

### 3.2 Suction rates

**Figure 2:**
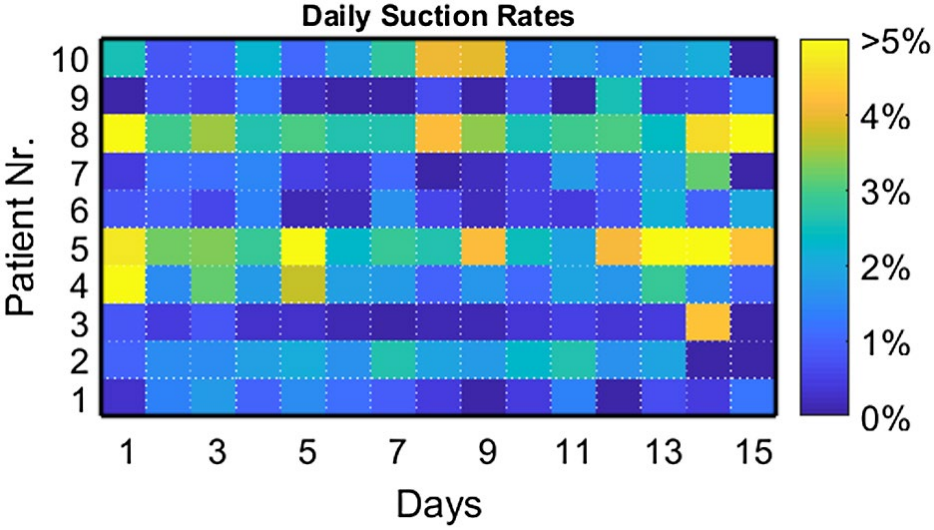
Daily suction rates in % per day: Heatmap chart of the individuals’ suction incidences calculated for every day of the observation period [Color figure can be viewed at wileyonlinelibrary.com]

**Figure 3:**
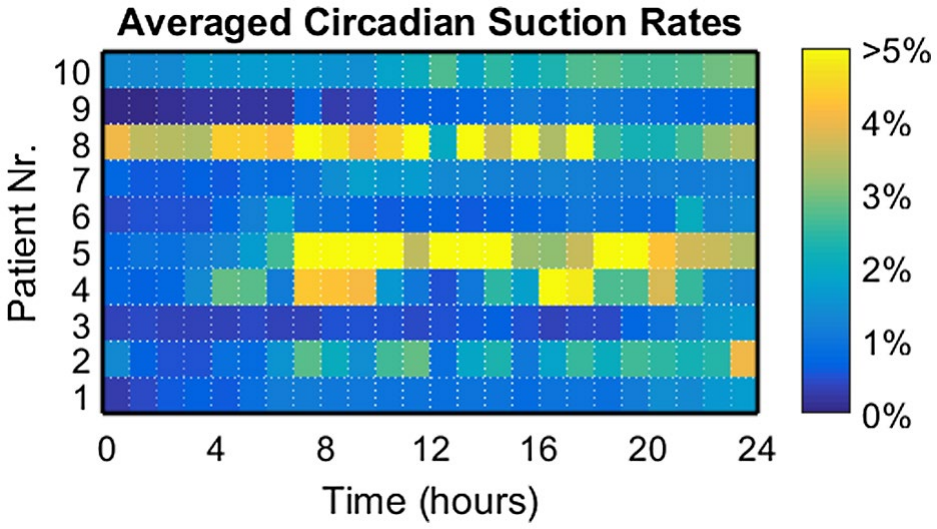
Suction rates per hour, averaged over the 15 days of the observation period [Color figure can be viewed at wileyonlinelibrary.com]

**Figure 4:**
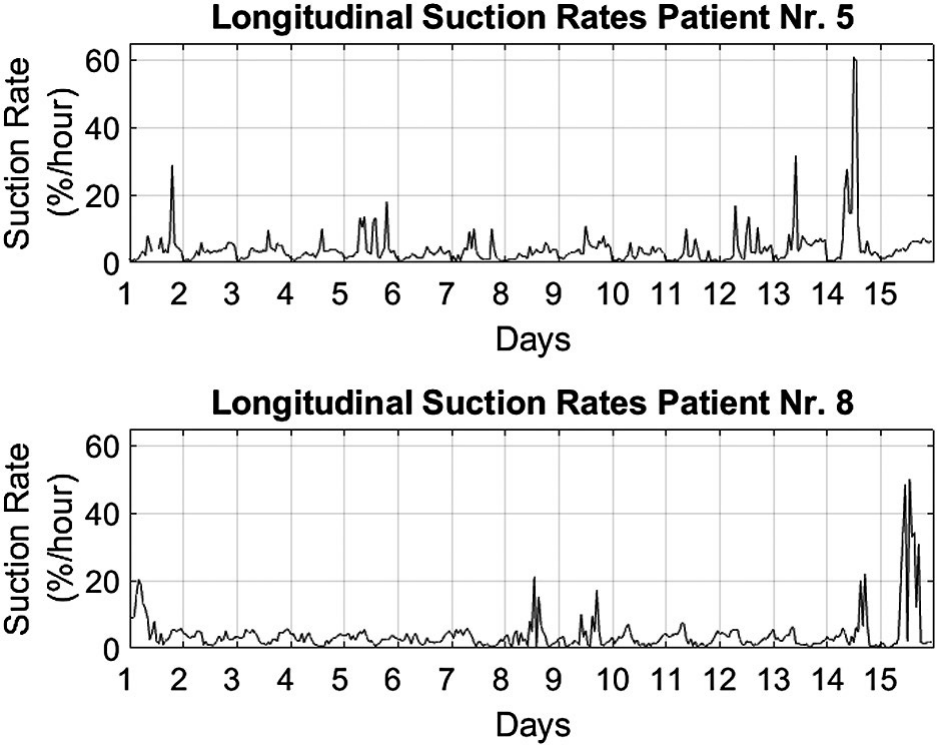
Longitudinal suction rates of the two patients (Nr. 5 and 8) with the most suction

From the recorded beats of all patients together, 1.1% of beats were classified as suction with a patient median of 0.8% and range 0.1%‐2.5% suction rates calculated over the whole observation period. One patient (Nr. 8) contributed 40% to the total suction beats, four patients (Nr. 2, 4, 5, and 10) 5%‐20% each (12.7 ± 4.5%) and five patients (Nr. 1, 3, 6, 7, and 9) less than 5% each (1.9 ± 1.2%). Figure 2 shows the individuals’ daily distribution of suction rates per patient. The large intraindividual variability of patients (Nr. 4, 5, and 8) necessitated saturation of the visualization at 5%. Figure 3 shows the individuals’ circadian suction rates per hour averaged over the 15 days. Longitudinal variation in suction rates per hour for the two patients with the highest suction rates are shown in Figure 4.

Individuals maximum suction rate per hour and occurrences of suction periods >5%/hour are shown in Table 2. For the comparison of patients with low and high suction rates two subgroups were compared (<10 vs >10 occurrences of suction rates >5%/hour). Comparison of the low‐suction group (Patient Nr: 1, 3, 6, 7, and 9) and the high‐suction group (Patient Nr: 2, 4, 5, 8, and 10) resulted in differences of the groups maximum suction rates (8% ± 5.3% vs 33% ± 22.8%, *P* = .04) and occurrences of relevant suction rates >5% (2 ± 2.1 vs 43 ± 26.0, *P* = .008). The patients' maximum suction rate per hour and the number of hours with >5% suction were highly correlated (*r* = 0.96, *P* < .0001).

**Table 2:** Individuals' maximum suction rates, occurrences of suction >5%/hour, and clinical data. (left ventricular diastolic diameter: LVEDD, left ventricular systolic diameter: LVESD)

Patient Nr	Maxi‐mum suction rate (% per hr)	Occurrence of suction rates >5% per hr (n)	Suction Group	Etiology of cardiomyopathy	Surgical access	Outflow graft location	LVAD Speed (rpm)	TTE Inflow cannula position	Number of ischemic Strokes (n)	Pump Thrombosis (y/n)	Late RV failure (y/n)	LVEDD (mm)	LVESD (mm)
1	3%	0	Low	Dilated	Sternotomy	Aorta ascendens	2700	Optimal	1	n	y	67	58
3	5%	0	Low	Dilated	Sternotomy	Aorta ascendens	3400	Optimal	0	n	n	71	64
6	17%	2	Low	Dilated	Bilateral thoracotomy	Subclavian artery	2600	Optimal	1	n	n	78	66
9	8%	3	Low	Ischemic	Bilateral thoracotomy	Aorta ascendens	2700	Suboptimal (suction)	0	n	n	59	57
7	7%	5	Low	Dilated	Bilateral thoracotomy	Subclavian artery	2700	Optimal	1	y	n	80	69
2	11%	12	High	Ischemic	Hemi‐Sternotomy & Thoracotomy	Aorta ascendens	2500	Optimal	1	n	n	48	44
10	10%	23	High	Ischemic	Sternotomy	Aorta ascendens	2800	Suboptimal	0	n	y	51	42
4	31%	44	High	Ischemic	Bilateral thoracotomy	Aorta ascendens	3000	Suboptimal (suction)	0	n	y	46	31
8	50%	61	High	Ischemic	Bilateral thoracotomy	Subclavian artery	2800	Optimal	5	y	y	54	45
5	61%	75	High	Dilated	Bilateral thoracotomy	Subclavian artery	2700	Suboptimal	2	n	n	68	61

### 3.3 Comparison of clinical measures for low‐ and high‐suction group

In the high‐suction group, heart failure etiology was primarily ischemic compared to the low‐suction group with more dilated cardiomyopathy (see Table 2). Similarities existed in operation technique (bilateral thoracotomy n = 3) and outflow graft anastomosis (aorta/subclavian artery: n = 3/2) among the two groups. Patients LVAD speeds ranged from 2500 to 3400 rpm with no statistical differences according to the suction group. LDH measured at the beginning of observation period was 269 ± 65 U/L and proBNP levels were 3.8 ± 2.7 ng/mL. Despite the occurrences of suction, efforts to compare lab parameters (LDH, fHb, bilirubin, and proBNP) and readmission profiles for the low‐ and high‐suction subgroups did not result in any relevant differences. In the low‐suction group, four patients had optimal inflow cannula position and one patient suboptimal position with the occurrence of suction during transthoracic echocardiography. In the high‐suction group, two patients had optimal inflow cannula position and three patients suboptimal position, with the occurrence of suction during echocardiography in one patient of the suboptimal group. Patients in the high‐suction group had significant smaller left ventricular end diastolic diameters (*P* < .018) as well as left ventricular end‐systolic diameters (*P* < .019). The transthoracic echocardiographic measurements were performed on median POD 128 and ranged 32‐227. The low‐ compared to the high‐suction group had the same prevalence of stroke (n = 3 vs n = 3, all ischemic strokes) with the first occurrence of stroke at median POD 164 and ranged from 1 to 267 days. The number of stroke events for each patient is shown in Table 2. For one patient in each group, pump thrombosis was diagnosed. Late right heart failure after the observation period occurred in one patient from the low‐suction group and three patients from the high‐suction group at median POD 702 days and ranged from 234 to 1487 days.

### 3.4 Suction bursts

**Figure 5:**
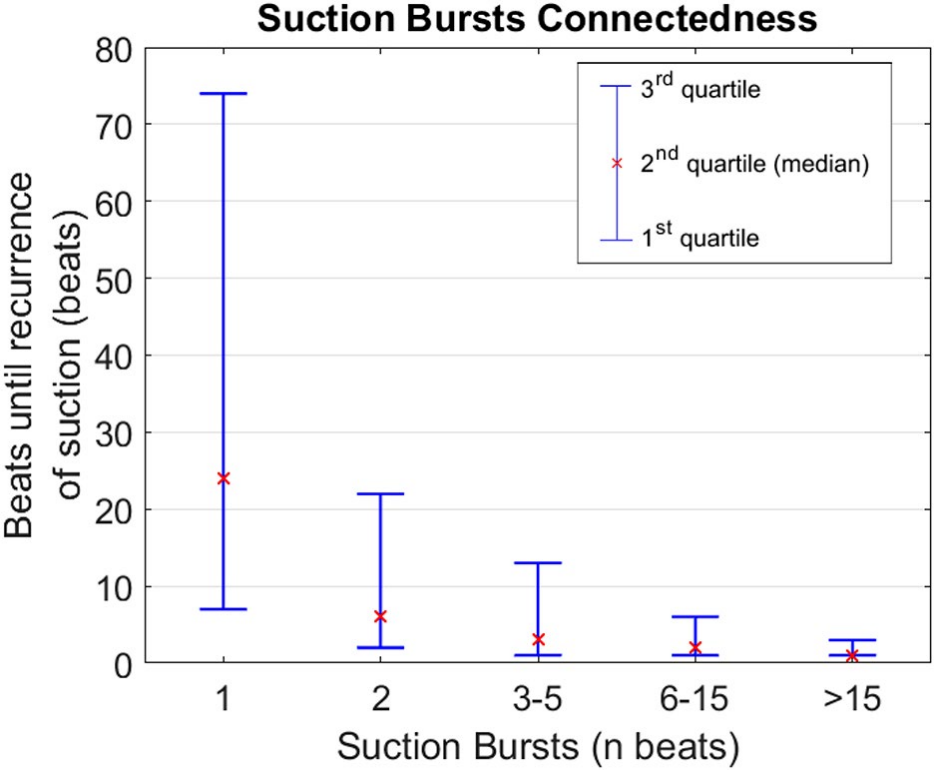
Extended suction bursts correlated with earlier recurrence of suction [Color figure can be viewed at wileyonlinelibrary.com]

Of all suction beats detected, 59% occurred as single events between nonsuction beats, 10.7% occurred as two consecutive suction beats, 9.8% occurred within bursts of 3‐5 consecutive suction beats, 9.4% within bursts of 6‐15 beats, and 11.1% with bursts of >15 consecutive suction beats. Longer bursts were correlated with shorter event‐free time periods after the burst had ended, as shown in Figure 5.

### 3.5 Clusters of suction events and correlated tachycardia

A total of 11 821 suction clusters were evaluated. Table 3 shows the distribution of durations and occurrences for suction clusters and tachycardia within these clusters. Independent of suction cluster duration within the 10‐second period before suction, tachycardia occurred with a median of 0.6% (range 0%‐3.8%) and 0.5% (range 0%‐6.7%) for the 10‐second post‐suction period. Tachycardia events were detected in nine patients with a patient median of 23.1 minutes (range 0.2 minutes‐39.8 hours). The patients' percentages of tachycardia during suction clusters showed a distribution with five patients well below 50% and four patients well above 50%. The five of the nine patients had only 14.4% (1.2%‐21.7%) of their tachycardia during suction clusters, whereas the other four patients had 93.6% (77.8%‐100.0%) of their tachycardia during suction clusters. The four patients with higher individual probabilities of tachycardia during suction had less tachycardia in general with a median duration of 5 minutes (0.2‐105.6 minutes) vs 54.1 minutes (range 21.6 minutes‐39.8 hours) in the 5 patients with lower probabilities (*P* = .19).

**Table 3:** Suction clusters analyzed by durations. Patient median and range are shown for clusters per patient, average duration per patient, suction incidence rate, and the tachycardia associated parameters

	Suction cluster duration		
	≤1 min	>1 min & ≤5 min	>5 min
Total clusters (n)	11 435	356	30
Occurrence in n patients	10 (100%)	8 (80%)	4 (40%)
Clusters per patient (n)	802.5 (42‐3737)	22.5 (3‐140)	6.5 (1‐16)
Cumulative Duration (hr)	44.6	10.0	4.6
Average cluster duration per patient (min)	0.2 (0.1‐0.3)	1.6 (1.3‐1.8)	9.3 (6.4‐10.3)
Suction rate within the cluster (%)	34.6% (27.1%‐44.7%)	41.4% (25.2%‐84.3%)	59.1% (52.8%‐91.4%)
Tachycardia occurrence (% of suction clusters)	3.4% (0.0‐18.1)	50.0% (0.0‐88.7)	100.0% (0.0‐100.0)
Tachycardia within cluster (% of total beats within cluster)	40.0% (13.4‐100.0)	14.8% (10.6‐30.3)	10.2% (2.8‐20.0)

**Figure 6:**
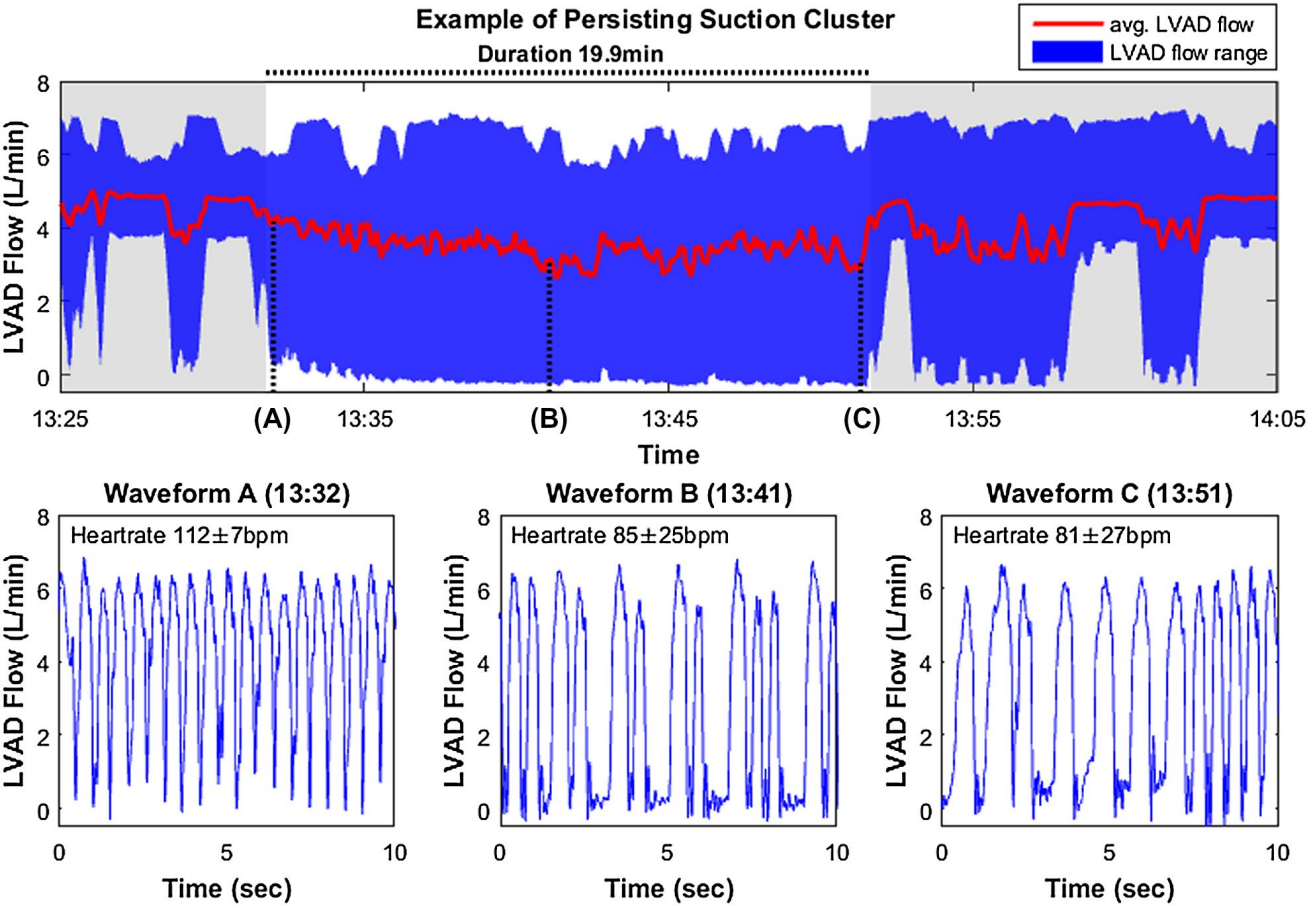
The cluster with the longest duration (19,9 min, occurred in Patient Nr. 5). Suction with tachycardia (A), followed by arrhythmia with waveforms indicating LV collapse (B and C) [Color figure can be viewed at wileyonlinelibrary.com]

In Figure 6, the longest suction cluster with 19.9‐minute duration and snapshots of the pump flow waveforms from Patient Nr. 5 are shown.

**Figure 7:**
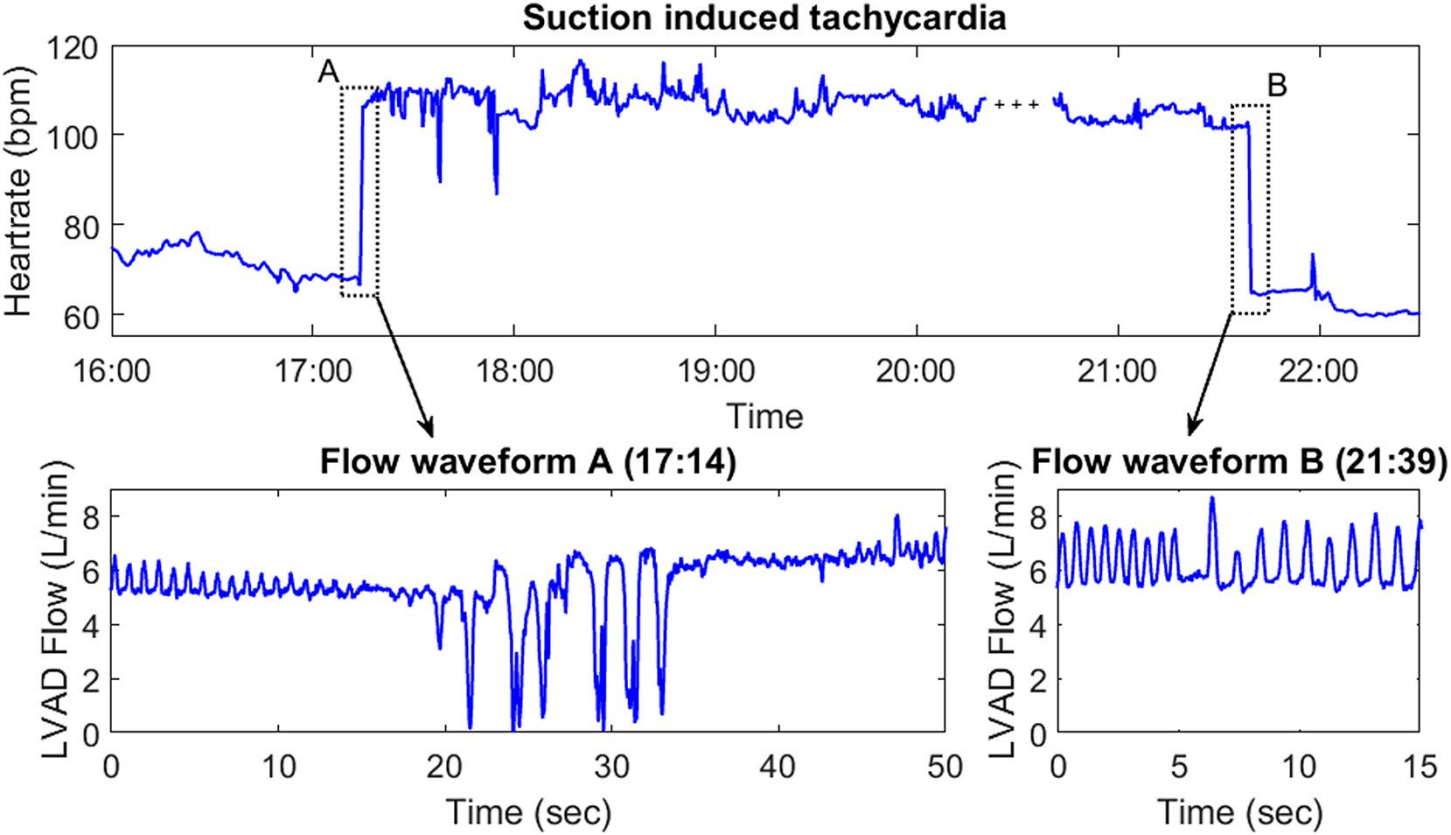
Tachycardia interval of 4.4 h triggered by a brief suction period (+symbol indicates missing data due to temporary disconnection from the LVAD data recorder, Patient Nr. 3) [Color figure can be viewed at wileyonlinelibrary.com]

A noteworthy observation is shown in Figure 7. Following normal hemodynamic conditions, the venous return (indicated by LVAD flow pulsatility) decreased temporarily leading to a burst of suction which triggered a long‐lasting tachycardia episode of 4.4 hours.

## 4 DISCUSSION

There is a broad consensus in the LVAD community that suction can increase risk for cardiac tissue damage, hemolysis, thrombus release, and abnormal heart rhythms.^6^, ^8^, ^9^, ^10^, ^11^, ^12^, ^13^ To the authors' knowledge, this study is the first comprehensive analysis of longitudinal suction occurrences from beat‐to‐beat interrogation of the pump in LVAD outpatients. An unanticipated high rate of unrecognized suction events occurred in half of the patients despite the initial assumption that they were optimally adjusted to their constant speed LVAD and medical therapy. Currently, only scarce definitions regarding the recognition and quantification of suction events in LVAD patients exist which are agreed upon by the LVAD society, presumably due to the limited monitoring capabilities. Three different measures to quantify and describe patients' suction behavior were reported, enabling acquisition of information regarding distribution of suction in LVAD outpatients: suction rates, suction bursts, and suction clusters.

Patients were assigned to a low‐ or high‐ suction group based on the rate of suction events that were observed, with a threshold of more than 5% per hour being considered a relevant suction rate. The threshold of 5% per hour was defined based upon the observed patients' maximum suction rates per hour. This provided a useful parameter for longitudinal data that is also feasible for patient stratification. Comparison of the two suction groups from early outpatient LVAD data resulted in significant differences in left ventricular systolic and diastolic diameters measured by echocardiography. Differences in etiology (ischemic vs non‐ischemic cardiomyopathy) were observed, albeit without statistical significance. Results from blood sample testing, tracking of adverse events, site of outflow graft anastomosis, and LVAD speed were comparable among groups. Confounding factors might be present based on these observations, therefore analysis in a larger patient cohort with adjustment for possible confounding factors is required.

Another clinically relevant observation is the association of suction and tachycardia, which had been initially reported for in‐patient observations in Vollkron et al.^11^ In this study, this association was further explored and refined in the continuous outpatient LVAD recordings, covering an observation period of 15 days. The results showed that ≥50% of suction clusters with >1 minute of duration were associated with tachycardia within the cluster. Immediate presuction or postsuction tachycardia occurred less frequently. The identification of the origin of abnormalities in cardiac rhythm (whether induced by suction or not) in the individual patient has implications on therapeutic adjustments, suggesting either treatment of electrically induced arrhythmia or prevention of suction by pump settings or fluid balance adjustments. Patient Nr. 5 is a clear example for this problem: The patient had the second highest number of overall suction beats and the longest identified suction cluster of all patients (see Figure 6). In this patient, 87.2% of a total of 106 minutes of tachycardia occurred during suction periods. Another patient (Nr. 3) had the least occurrence of suction of all patients, however, a short suction event stimulated long‐lasting tachycardia (see Figure 7). This patient had only one additional tachycardia episode consisting of five beats within the observation period. With speed responsive LVADs these suction related rhythm abnormalities could likely be avoided and therapy could in turn be optimized accounting for the hemodynamic fluctuations shown in Figure 4.

To understand the mechanistic principles of the suction induced tachycardia, it is important to highlight that the mechanical irritation of the cardiac tissue by a single suction beat can be a potent stimulus triggering an ectopic ventricular contraction via mechano‐electric coupling.^35^ The insufficient left ventricular filling leading to suction and the concomitant‐induced ectopic ventricular contraction can start a vicious cycle of suction with induced tachycardia in this hemodynamic situation.^11^

In some LVAD patients, pacemaker therapy is adjusted to higher rates in an effort to reduce afterload of the weak right ventricle to prevent right heart failure. Meanwhile, reducing preload on the left ventricle in this hemodynamic situation may lead to initiation of suction events. An example from our observations is Patient Nr. 8, who had by far the most occurrence of suction as well as occurrences of tachycardia. Individual analysis of this patient showed frequent occurrences of longer suction periods connected to the high heart rates at rest. In this patient, the clearance of suction occurred often due to an increase in venous return caused by walking, which was shown through analysis of the triaxial accelerometer data within the LVAD data recorder.

Independent of cardiac rhythm it is clear that any solid structure within the left ventricle or the atrium that cannot resist the local low pressure generated by the LVAD will eventually result in suction events. The likelihood of this form of suction event occurring is increased with inflow cannula malposition as mentioned in reference.^35^ The conditions leading to this type of suction are different to those of volumetric suction which has been the focus of the discussion so far. Volumetric suction events occur during periods of low preload, which very often appear after a decline in pump flow pulsatility, similar to Figure 7 (Waveform A). For severe inflow cannula malposition, either due to device implantation or reverse remodeling of the left ventricle, this type of suction occurs with near normal left ventricular filling and preload. In case of suboptimal cannula positioning, suction events do not always occur with the aforementioned preceding decline in pump flow pulsatility. Furthermore, suction events associated with cannula malposition show waveforms consisting of unconventionally large pulsatilities, clearly representing the systolic portion of the cardiac cycle, as observed in the patients’ LVAD data. It is worth noting that Patient Nr. 6 had multiple episodes of collapsing due to orthostatic problems prior to the observation period during rehabilitation. Analysis of the LVAD data recordings during these episodes showed reduced left ventricular filling and consequently excessive suction events leading to rhythm disorders with adequate ICD shock responses (analyzed retrospectively with the ICD logs together with LVAD data). The improvements in LVAD device settings and fluid balance (without the need to increase the dose of anti‐arrhythmia drugs) lead to a complete disappearance of such episodes, with low numbers of suction episodes after adjustment (see Table 2).

As the aforementioned example shows, LVAD therapy must be tailored to the particular patient's condition and is highly individual.^4^ With the continuous monitoring used in this study it is possible to identify the unique hemodynamic LVAD fingerprint and analyze its progression over time. While a single snapshot during the outpatients’ examination is not sufficient for such an analysis. Suction events can be determined through echocardiographic imaging, Valsalva maneuvers, right heart catheterization among other methods by healthcare professionals to estimate the likelihood of suction occurrence and to confirm suspected suction events. Strategies to reduce the occurrence of suction events are LVAD speed reduction and patient education to manage fluid uptake and diuretics therapy as well as the self‐assessment of LVAD parameters multiple times a day and during symptomatic periods. The ability to perform an analysis of pump performance coupled with the ability to detect pathological changes in the pump's operating conditions is unprecedented and could potentially be implemented as a high‐fidelity monitoring tool in the prospective management procedures of the pump. Data collected with this unique technology is one of the many steps required in the development of future generations of “smart‐pumps.”

The inability of healthcare professionals to retrospectively examine suction alarm events with current clinical LVAD monitoring impedes the identification of alarm performance and prevents proper individual adjustments of alarm thresholds. Two current generation devices, the Incor (Berlin Heart, Berlin, Germany) and the Heartmate 3 (Abbott Laboratories, Abbott Park, IL, USA), contain mechanisms for temporary reduction in LVAD speed in case of detection of suction events. The performance of these detection algorithms and the efficacy of their suction clearance strategies have not yet been reported, to our knowledge.

As the observations and analyses in this study show, improvements in LVAD monitoring to provide comprehensive information to the healthcare professionals are necessary. In terms of monitoring, LVADs can provide unique but limited sensory functions in addition to their main purpose, which is to deliver blood to the systemic circulation. LVAD monitoring can be useful not only to categorize suction patterns, but also to asses hemodynamic parameters of the assisted left ventricle such as aortic valve opening,^10^, ^20^, ^36^ contractility,^21^ relaxation,^22^ ventricular rhythm,^18^ and potentially even preload levels^37^, ^38^ or responses to different physical activities.^5^ Additional information such as data from pressure sensors or accelerometers in combination with LVAD data could further improve the quality of an individual's LVAD therapy.^39^ Modern monitoring could utilize telemonitoring to provide a sufficient connection with the healthcare professionals and to improve temporal resolution of monitored LVAD signals.^40^ Initial clinical experiences with telemonitoring in LVAD patients were reported previously.^41^

Although this study reports novel occurrences of suction events in stable LVAD outpatients, it has some limitations. The results are not capable of proving statistically significant differences in clinical endpoints of patients with low‐ and high‐suction rates. The number of selected patients is low and the observation window is too short to draw any definitive conclusion about potential effects of suction frequency on clinical adverse events or endpoints. Despite the low number of patients and short observation periods, this study shows that suction events occur in clinically stable outpatients and the comparable clinical parameters among the high‐ and the low‐suction group. Heart rate was not monitored by the gold standard ECG but derived from LVAD flow, which shows good accordance to ECG heart rates.^18^ The ground truth for the training and testing of the suction detection was based on visual observations of experts without additional use of echocardiography or invasive pressure measurements. For the outpatient monitored data, the ground truth for suction and nonsuction beats was inaccessible, which could provide important information due to the high occurrence of single isolated suction beats. The suction alarm of the HVAD was disabled in our patients (similar to other centers in Europe) due to numerous experiences of apparent false positive suction alarms. Therefore the clinically available alarm strategy implemented in the LVAD could not be compared and validated with the incidences identified with the continuous monitoring in this study. Echocardiography was not performed during the observation period and the comparison of the binary quantification of cannula position during examination with the suction occurrences over a duration of 15 days must be interpreted with caution.

## 5 CONCLUSION

Continuous LVAD monitoring revealed suction rates of outpatients with constant speed LVADs and optimal therapy, of which the healthcare professionals were unaware of. Five of the 10 studied patients showed multiple considerable suction events within the 15 days of study period. Longer suction episodes are more likely to initiate suction induced tachycardia. This study presents evidence to make the case for the implementation of a physiologic control concept to improve cardiac protection of LVAD patients.
